# Optical Chemical Sensors: Design and Applications

**DOI:** 10.3390/s23115284

**Published:** 2023-06-02

**Authors:** Roberto Pizzoferrato

**Affiliations:** Department of Industrial Engineering, University of Rome Tor Vergata, 00133 Rome, Italy; pizzoferrato@uniroma2.it

More than ever, optical chemical sensing is a thriving research field with a strong outlook in terms of future development and penetration into growing industrial markets. This situation is undoubtedly due to the peculiar characteristics of optical sensing, a methodology that can take advantage of a great variety of different effects and parameters, including fluorescence, light absorption, refraction, reflectance, optothermal effect, total internal reflection, surface plasmon resonance, interferometry, etc. All these effects can be sensitive to chemical interactions, and this continuously broadens the area of possible analytes. In addition, optical sensors can show high selectivity, immunity to electromagnetic interference, and safety while working with flammable and explosive compounds. They can easily be miniaturized, integrated with electronics to form lab-on-a-chip systems, and can also come in the form of cheap and disposable devices. Moreover, the combination of current familiar electronic devices with video cameras, such as smartphones, opens new perspectives for user-friendly optical sensing platforms that are available to citizens for onsite diffuse monitoring. These manyfold aspects are strongly conducive to the development of design and the search for new applications, as is well-represented in this Special Issue, which collects twelve high-quality papers. Three papers are systematic review articles and nine papers are original research articles. Numerous detection techniques with many different sensing materials and a vast range of chemical analytes are reported and investigated so as to give the current state of the technology in this exciting scientific topic.

The paper by Bendicho et al. [1] is an extensive and critical review on Paper-Based Analytical Devices (PADs) for the colorimetric and luminescent detection of mercury in waters. Sensing via PADs, also referred to as lab-on-paper technology, has recently received increasing attention and is likely to be key in the future for a broad range of applications, ranging from environmental monitoring to food safety, as well as medical diagnostics. In fact, PADs might be the answer to the demand for miniaturized, low cost, and fast analytical methods that allow a decrease in samples, reagents, and energy consumption. In the specific case, the review goes through the different materials that have been investigated for the sensing part of PADs. After considering the well-established organic chromogenic/fluorogenic receptors, much attention is paid to the variety of novel nanomaterials that have been introduced recently. Plasmonic nanoparticles (NPs), e.g., gold nanoparticles (AuNPs), silver nanoparticles (AgNPs), gold nanorods (AuNRs), and fluorescent nanoparticles, e.g., quantum dots (QDs), carbon dots (CDs), graphene quantum dots (GQDs), and metal nanoclusters (NCs), have been applied to build novel sensing assays. A critical discussion about shortcomings and outlook highlights the fact that the reported LODs are mostly in the ppm region, despite the required detection ability at the ppb level. In addition, problems could also arise when PADs based on nanoreceptors are used for real samples such as wastewater, seawater, and biological matrices. 

The authors of [2] performed an extensive and thorough review on optical fiber-based sensors for the measurement of ethanol, mainly in aqueous solution. Ethanol is one of the key chemicals used in a great number of modern industrial processes and consumer products. Due to this importance, it is not surprising that researchers have extensively investigated all the sensing strategies allowed by optical fibers, namely absorption, external interferometric, internal fiber grating, and plasmonic sensing. Fluorescence-based optical fiber and whispering gallery mode type sensors have also been reported in limited cases. This review critically analyses the advantages and disadvantages of the different methods in terms of sensitivity, selectivity, cross-sensitivity, compensation, and simplicity of manufacturing techniques. Attention is also paid to reusability and the need to target specific applications, as well as to the chance offered by distributed optical fiber sensing techniques. 

In Reference [3], Caroleo et al. systematically review recent progress in the field of optical chemical sensors for persistent organic pollutants (POPs), a broad class of chemicals that represent one of the greatest challenges for the environment and human health due to their resistance to degradation, significant adverse impacts, and capability to bioaccumulate in living tissues. The present, non-exhaustive list of POPs includes, but is not limited to, organochloride pesticides, polychlorinated biphenyls (PCBs), organophosphorus (OPs) pesticides, polyaromatic hydrocarbons (PAHs), flame retardants, and per- or poly-fluoroalkyl compounds (PFAS). Fluorescence is the main sensing technique for POPs, even though methods based on other optical parameters have also been developed, such as refractive index in Surface plasmon Resonance (SPR) and reflectivity in Surface Enhanced Raman Spectroscopy (SERS), absorbance in colorimetric sensors, biosensors and multisensor systems. Interestingly, within fluorescence sensing, due to the large chemical variety of POPs, different strategies based on turn-on, turn-off, and ratiometric effects have been explored. In addition, several methods for binding the target analyte with specific receptor sites are reported, including antibodies and aptamers, which are short, single-stranded DNA or RNA molecules that can selectively bind to a target with high affinity and specificity. This very broad field of research along with the technological advancements of familiar electronic devices, such as smartphones, etc., open new perspectives for the development of effective, inexpensive, and user-friendly sensing platforms.

The work by Rodrigues et al. [4] addresses the very contemporary topic of hybrid sensing materials with magnetic properties for simultaneous analyte detection and removal. In particular, the authors synthesized new unsymmetrical tri(tosylamino)phthalocyanines (Pc1) grafted to ferromagnetic silica nanoparticles (MSNPs). This new hybrid nanomaterial (MSNP-Pc1) formed stable dispersions of nanoparticles in water with optical colorimetric response to some anionic species such as CN−, F−, and OH−, which are some of the most common pollutants in residual and fresh waters. Interestingly, MSNP-Pc1 demonstrated complete reversibility with the addition of trifluoroacetic acid. This behavior, along with the sensitivity to external static magnetic fields, enabled recovery of the hybrid nanomaterial material and its reusability in a sensing process that was repeated five times without significant loss of anion sensibility. 

Turn-on of autofluorescence from NADH, which is a reduced form of the coenzyme β-nicotinamide adenine dinucleotide (NAD^+^), was explored by Toma et al. [5] to detect methanol (MeOH) in exhaled breath using a biochemical gas sensor (bio-sniffer) with the potential for non-invasive assessment of intestinal flora. The method was based on a cascade reaction with two enzymes, alcohol oxidase (AOD) and formaldehyde dehydrogenase (FALDH), as follows: oxidation of MeOH was initially catalyzed by AOD to produce formaldehyde, and then formaldehyde was successively oxidized via FALDH catalysis together with the reduction of oxidized NAD^+^. This cascade reaction thus produced a reduced form of NAD (NADH) proportionally to the concentration of MeOH and NADH detected by exciting its autofluorescence with UV light. The method might seem quite indirect and complicated, but this peculiar cascade reaction enabled specific detection of MeOH with high selectivity, especially from other aliphatic alcohols including ethanol, 1-propanol, 1-butanol, and 2-propanol. In addition, it has the advantage of being quite unsensitive to the relative high humidity (65–91%) of exhaled breath. The bio-sniffer showed a linear range of 0.32–20 ppm, which encompasses the typical values of MeOH concentration in exhaled breath (0.10–2.3 ppm).

A low-cost plastic lab-on-a-chip platform for the automatic continuous monitoring of nitrites in water for aquaculture farming is reported in Reference [6]. The detection of nitrites is carried out through colorimetric measurements by using the well-known Griess reaction. This is a clear example of the level of automatization of analytical analysis that can be achieved by utilizing microfluidics and well-known reliable assays to perform many repeated measurements with limited amounts of reagents. In particular, the authors designed and manufactured a six-layer poly (methyl methacrylate) transparent assembly where reagents and water samples are mixed. The color change was read by a simple and cheap integrated optoelectronic setup that could reveal nitrite concentration with an LOD of about 2 µM. Even though this value is higher than that achieved with other methods using the Griess reaction, it is sufficient for aquaculture purposes and was obtained with low-cost components.

The authors of [7] report on naked-eye detection of morphine through a novel and facile colorimetric assay based on silver citrate-coated Au@Ag nanoparticles (Au@AgNPs). Optical excitation of the surface plasmon resonance of gold or silver nanoparticles is currently used as a sensing strategy for a variety of analytes. However, in this work, Au@Ag NPs were synthesized using a simple and green sonochemical approach using either high- or low-power ultrasonic irradiation for the first time. The optimized sonochemical method resulted in a simple and fast sensing procedure that produced clearly visible color changes due to nanoparticles’ aggregation in less than 5 min. LODs for morphine of 0.100 µg/mL and 0.055 µg/mL were obtained with high- and low-power ultrasonic synthesis, respectively. The linear range was 0–50 µg/mL and 0–30 µg/mL, respectively.

In Reference [8], Viola et al. report an innovative solution to improve Quartz-Enhanced Photo-Acoustic Spectroscopy (QEPAS) for gas/vapor sensing. The authors used additive manufacturing to build the absorption detection module monolithically using micro-metal laser sintering. The proposed method permitted the detection of ammonia in nitrogen with an LOD better than 1 ppm and, for its characteristics, paves the way for a new family of portable sensors for the identification of complex solids, such as sticky compounds with a high boiling point, and liquid traces samples, including compounds with low volatility such as illicit drugs, explosives, and persistent chemical warfare.

The optical sensing properties of carbon dots towards some heavy metal ions in water are reported in References [9,10,11]. Carbon dots (CDs), also referred to as graphene quantum dots (GQDs), are a class of carbon nanoparticles that have recently attracted much attention for their sensitivity to heavy metals (HMs). The optical response shows through both fluorescence and absorption colorimetric variations and is mainly due to the presence of different oxygen-containing functional groups on the main carbon structure that strongly interact with HMs. This presence can be partially tuned by appropriate synthesis and doping procedures. In fact, in Reference [9], CDs were produced using a strongly oxidative method for the cage opening of C_60_ fullerene and they demonstrated a visible colorimetric response to Cr(VI). On the other hand, nitrogen-doped CDs synthesized starting from o-phenylenediamine (OPD) through a hydrothermal method [10] presented a colorimetric response that could be tuned via pH regulation to achieve multiple sensitivity to Cu(II) and Co(II) in different regions of the visible spectrum. Due to the presence of bound OPD, the same CDs also showed a fluorescence enhancement [11] that was visible after incorporation incorporated in a hydrogel agarose matrix for easier handling and application.

Very often, optical sensing must face light transmission through “turbid media”, where light absorption and scattering effects are mostly observed together, and this can impact the accurate detection of fluorescence and colorimetric responses. In Reference [12], Ducanchez et al. used an original approach that mixes polarized light spectroscopy and the Mueller matrix concept coupled with partial least square calibration models. This allowed the authors to demonstrate that absorption and scattering effects can be distinguished in the Rayleigh regime with linear and circular polarization from the M_22_ and M_44_ elements of the Mueller matrix, respectively. 

In summary, we believe that this Special Issue highlights some of the most representative lines of a topic as broad as sensing strategies, with a large range of possible approaches, many different methods of implementation, and a broad and expanding range of sensing materials and analytes. This combination is key for the continued progress of design and the growth of present and potential applications. The present articles prove this present situation and allow the considering of optical chemical sensing as one of the most interesting and versatile sensing methodologies.
